# The efficacy of furmonertinib in untreated advanced NSCLC patients with sensitive EGFR mutations in a real-world setting: a single institutional experience

**DOI:** 10.3389/fonc.2024.1331128

**Published:** 2024-02-22

**Authors:** Ningning Yan, Sanxing Guo, Siyuan Huang, Huixian Zhang, Xingya Li

**Affiliations:** Department of Oncology, The First Affiliated Hospital of Zhengzhou University, Zhengzhou, Henan, China

**Keywords:** furmonertinib, non-small cell lung cancer, EGFR-mutated, epidermal growth factor receptor, real-world setting

## Abstract

**Background:**

Furmonertinib is the standard treatment option in the first-line setting for advanced non-small cell lung cancer (NSCLC) with sensitive epidermal growth factor receptor (EGFR) mutations in China. However, there are limited real-world data available.

**Methods:**

We conducted a retrospective study at a single center, analyzing a cohort of 73 NSCLC patients who tested positive for EGFR mutations and were treated with furmonertinib as their initial therapy between August 2022 and December 2023. The primary endpoint was progression-free survival (PFS), with secondary endpoints including objective response rate (ORR), overall survival (OS), and safety profile.

**Results:**

The median observation period was 9 months (95% confidence interval [CI], 8.0–20.0). The median PFS was 19.5 months (95% CI, 14.6–24.4). OS data were not yet mature. Univariate analysis showed no significant correlation between PFS and factors such as Eastern Cooperative Oncology Group performance status (ECOG PS) score, presence of brain or liver metastases, sex, age, EGFR mutation status, or number of metastatic sites. However, multivariate analysis indicated a potential trend toward extended PFS in patients younger than 65 years (p = 0.053, 95% CI, 0.10–1.02), although the p-value was only marginally significant. The most common adverse events were diarrhea (24%), anemia (36%), and liver injury (32%); however, only four cases experienced severe adverse events.

**Conclusion:**

In a real-world setting, furmonertinib appears to be a favorable treatment option for EGFR-mutated patients. The manageable nature of adverse events further supports its use in clinical practice.

## Introduction

Lung cancer is the leading cause of cancer-related deaths worldwide, with a mortality rate of 18% (1). In China, lung cancer is also the most common cancer in terms of incidence and is the leading cause of cancer-related mortality (2). Among the different subtypes of lung cancer, non-small cell lung cancer (NSCLC) is the most frequently diagnosed histological subtype at initial diagnosis, with adenocarcinoma being the most common subtype. Approximately 60% of NSCLC patients with adenocarcinoma harbor oncogenic driver mutations, with the epidermal growth factor receptor (EGFR) mutation being the most commonly found and targetable driver mutation in NSCLC (3).

Current standard treatment for NSCLC patients with EGFR-sensitive mutations, such as exon 19 deletion (19DEL) and substitution of lysine with arginine in exon 21 (21L858R), involves the use of EGFR–tyrosine kinase inhibitors (TKIs). These TKIs have been shown to prolong the survival of EGFR-mutated NSCLC patients (4–10). Currently, there are three generations of EGFR-TKIs approved for use in EGFR-mutated NSCLC. In China, a total of eight agents targeting EGFR mutations are approved. First-generation EGFR-TKIs have demonstrated an objective response rate (ORR) of approximately 60%–80% and a median progression-free survival (mPFS) of 8–13 months (4, 5, 10, 11). Second-generation agents targeting EGFR mutations have shown an extended mPFS of 11–16.5 months (6, 12). The third generation of EGFR-TKIs, including osimertinib, almonertinib, and furmonertinib, have shown even better efficacy with an mPFS of 18.9–20.8 months (7–9). Due to their improved efficacy and ability to penetrate the brain, third-generation agents are now recommended as the preferred treatment option for EGFR-mutated NSCLC.

Furmonertinib, a third-generation EGFR-TKI, is an original drug developed by a Chinese pharmaceutical company. Furmonertinib is a potent irreversible inhibitor of EGFR that specifically targets mutations in the receptor. It is effective against two types of mutations: resistance mutations (T790M) and activating mutations (L858R and exon 19 deletions). Furmonertinib is more effective at inhibiting tumor cells with these mutations compared to cells with the normal, wild-type EGFR. In the FURLONG study, a randomized Phase III trial that included previously untreated advanced NSCLC patients with EGFR-sensitive mutations, furmonertinib demonstrated superior PFS compared to gefitinib (20.8 versus 11.1 months, hazard ratio [HR] 0.44, 95% confidence interval [CI], 0.34–0.58, p < 0.0001) (9). The PFS achieved with furmonertinib was numerically longer than that observed with other third-generation EGFR-TKIs such as osimertinib and almonertinib, making it the longest achieved PFS to date. It is worth noting that the FURLONG study was conducted in China, exclusively including Chinese NSCLC patients. This suggests that furmonertinib may be particularly well-suited for Chinese NSCLC patients with EGFR mutations. However, the superiority of furmonertinib in terms of overall survival (OS) remains unreported due to the immaturity of OS data. Thus, real-world evidence regarding furmonertinib as a first-line treatment option for EGFR-mutated NSCLC patients, as well as clinically measurable prognostic factors, remains limited.

The objective of this report is to explore real-world data on the efficacy and safety of furmonertinib as a first-line treatment option in routine clinical practice.

## Patients and methods

### Study design

This retrospective study was conducted at a single center with the objective of examining the effectiveness of furmonertinib in patients diagnosed with previously untreated NSCLC harboring EGFR mutations. The study included patients treated at the First Affiliated Hospital of Zhengzhou University between October 1, 2021, and July 19, 2023.

### Patients

Consecutive cases of advanced/metastatic NSCLC with EGFR mutations, who received furmonertinib as their initial treatment between October 2021 and July 2023 at the First Affiliated Hospital of Zhengzhou University, were included in this retrospective study. The data cutoff date was September 24, 2023. The key inclusion criteria were as follows: 1) patients aged 18 years or older; 2) patients pathologically confirmed with unresectable locally advanced (stage IIIB/C: unfit for radical surgery or local radiotherapy) or metastatic (stage IV) NSCLC with EGFR actionable genomic mutations according to the TNM staging system, American Joint Committee on Cancer (AJCC) 8th edition; 3) no previous treatments given, with a treatment interval of at least 12 months after radical mastectomy for those who received neoadjuvant or adjuvant therapies; 4) presence of at least one measurable target lesion based on Response Evaluation Criteria in Solid Tumors, version 1.1 (RECIST v1.1) (13); 5) expected survival of over 3 months. Key exclusion criteria were as follows: 1) concurrent receipt of other anticancer treatments or any previous anticancer treatments prior to furmonertinib administration; 2) tumors mixed with small cell lung cancer (SCLC) components; 3) allergy to furmonertinib or its metabolic product; 4) history of interstitial pulmonary disease (IPD) or any uncontrolled/severe complicating comorbidity. Clinicopathological variables such as age, sex, Eastern Cooperative Oncology Group performance status (PS), smoking status, histology, stage, metastatic organs, EGFR mutation types, concomitant mutations, and programmed death-ligand 1 (PD-L1) expression were retrospectively collected from documented health records. The present report obtained approval from the Ethics Committee of the First Affiliated Hospital of Zhengzhou.

At the start of the study, tests for EGFR mutations were performed using either real-time polymerase chain reaction (PCR) or next-generation sequencing (NGS). For evaluating PD-L1 expression, tumor samples were analyzed using immunohistochemistry (IHC) with the DAKO 22C3 PharmDx antibody (Dako, Carpinteria, CA, USA). The levels of PD-L1 were determined by the tumor proportion score (TPS), which measures the percentage of positive tumor cells.

### Intervention

Patients received furmonertinib 80 mg orally once daily until disease progression or severe or unmanageable toxicities were developed.

### Outcome measure

The primary endpoint was PFS, as assessed by the investigators using RECIST 1.1 criteria. Key secondary endpoints included OS, ORR, disease control rate (DCR), and safety data. The ORR encompassed the percentage of patients achieving either a complete response (CR) or a partial response (PR) to the therapy. The DCR was defined as the number of patients who experienced a CR, a PR, or a stable disease (SD). PFS was defined as the duration from the first dose of furmonertinib until progressive disease (PD) or death. OS was calculated as the time from the start of treatment until death from any cause. Patients were considered censored if they were still alive at the time of their last recorded visit.

### Safety

Safety data were collected whenever adverse events (AEs) led to modifications in treatment or serious adverse events (SAEs) occurred.

### Statistical analysis

Statistical analyses were carried out using SPSS version 21.0 (IBM Corp., Armonk, NY, USA), and all charts were created using GraphPad Prism version 8.0. Descriptive statistics were applied to all variables where appropriate. Continuous variables were presented with the number of patients, median, and range (minimum and maximum values). Categorical variables were shown as frequency counts and percentages. A p-value of less than 0.05 was considered significant. Survival curves were generated using the Kaplan–Meier method, and survival analysis was conducted using a stratified log-rank test. HRs and 95% CIs were calculated using Cox regression analysis.

## Results

### Patients’ clinicopathological characteristics

**Figure 1:**
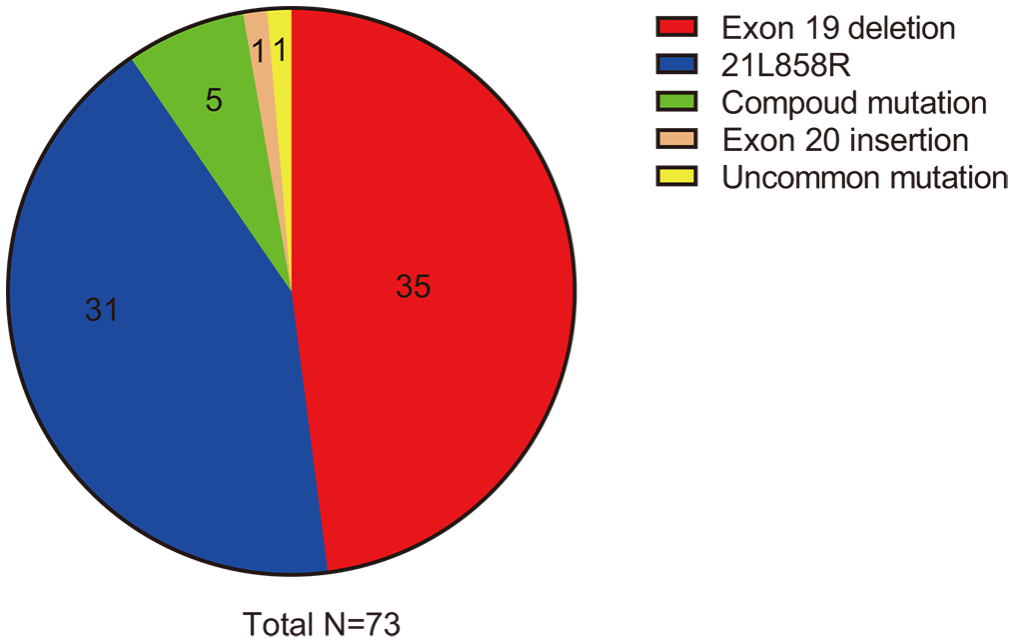
EGFR mutation types included in the study. EGFR, epidermal growth factor receptor.

From October 1, 2021, to July 19, 2023, we performed a retrospective review of 73 patients with advanced EGFR-mutated NSCLC diagnosed at the First Affiliated Hospital of Zhengzhou University. Out of these patients, a total of 32 (43.8%) were found to have brain metastases at the time of initial diagnosis, with an additional two patients presenting leptomeningeal metastases when first diagnosed. The majority of patients (69 out of 73, or 94.5%) had adenocarcinoma, while two patients had squamous cell carcinoma, and two had adenosquamous carcinoma. Notably, 35 patients (47.9%) were male, and 16 patients (21.9%) were current or former smokers (with quitting time less than 15 years). The median age of the group was 61 years (ranging from 30 to 85 years), which is consistent with the typical clinical profile of patients with EGFR-mutated NSCLC. The EGFR exon 19 deletion mutation occurred in 35 patients and the L858R point mutation in 31 patients. Two patients exhibited primary resistance mutations (T790M point mutation in exon 20) to first- or second-generation EGFR TKIs, and compound mutations were observed in five patients, including one with a rare G719A/S768I mutation (as shown in **Figure 1**). Additionally, co-occurring TP53 mutations were found in seven patients, with one patient also having concurrent EGFR 21L858R, TP53, and RET–IGR fusion mutations. Other details are summarized and presented in **Table 1**.

**Table 1:** Baseline clinicopathological characteristics.

Parameters	N (%)
Total patients	73 (100.0)
Median age (range), years	61 (30–85)
Gender Male Female	35 (47.9) 38 (52.1)
Smoking history No Yes	52 (71.2) 21 (28.9)
Histology Adenocarcinoma Squamous Adenosquamous	69 (94.5) 2 (2.7) 2 (2.7)
Stage IV	73 (100)
ECOG PS 0 1 2	19 (26.0) 46 (63.0) 8 (11.0)
Mutation status 19DEL 21L858R Rare Compound	35 (47.9) 31 (42.5) 2 (2.7) 5 (6.8)
Metastatic sites <3 ≥3	58 (79.5) 15 (20.5)
Brain metastases Yes No	32 (43.8) 41 (56.2)
Liver metastases Yes No	4 (5.5) 69 (94.5)
Bone metastases Yes No	36 (49.3) 37 (50.7)
Adrenal metastases Yes No	6 (8.2%) 67 (91.8)

ECOG PS, Eastern Cooperative Oncology Group performance status.

### Treatment efficacy

**Figure 2:**
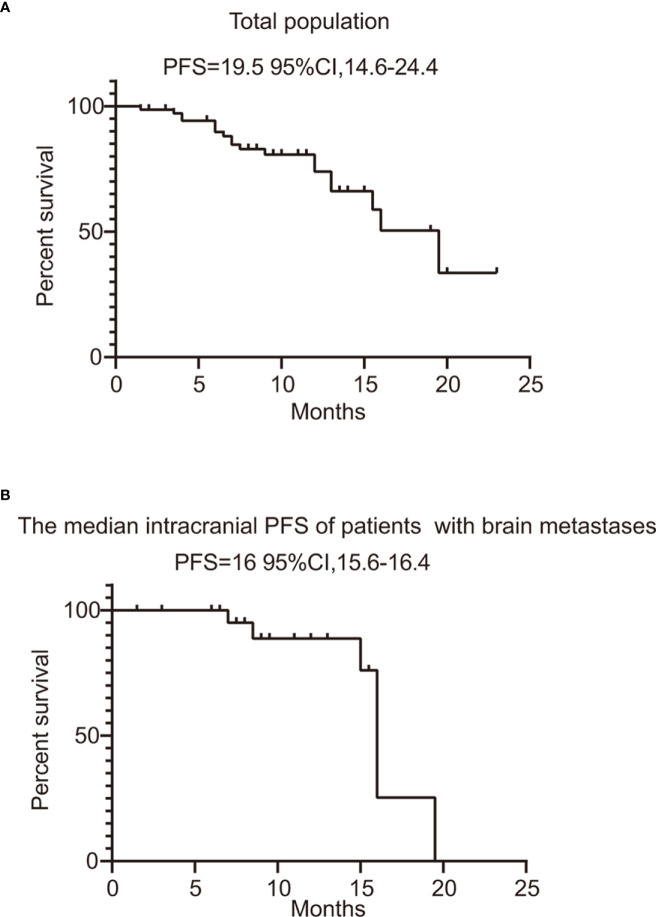
PFS of total population and patients with evaluable brain metastases. **(A)** PFS of all the patients treated with furmonertinib. **(B)** the median intracranial PFS of patients with brain metastases treated with furmonertinib. PFS, progression-free survival.

**Figure 3:**
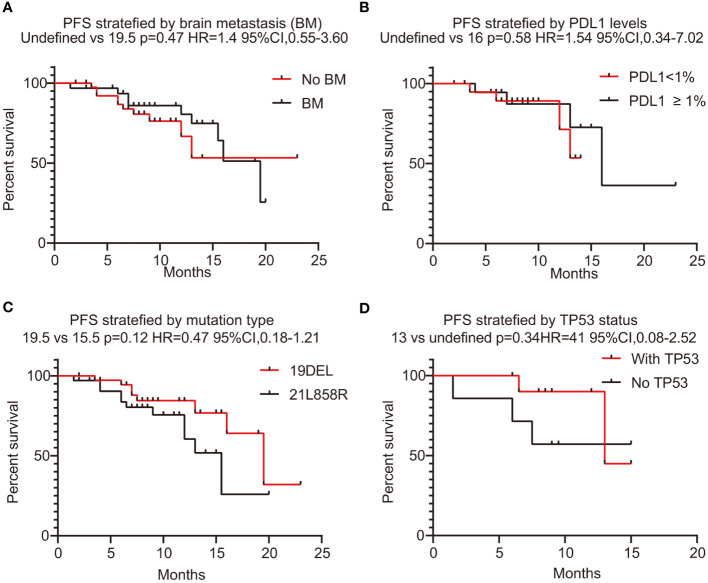
PFS stratified with brain metastases, PD-L1 expression level, EGFR mutation status, and co-mutation TP53. **(A)** PFS stratified with brain metastases (yes or no). **(B)** The correlation between PFS and PD-L1 levels (<1% *vs.* ≥1%). **(C)** The correlation between PFS and mutation type (19DEL *vs.* 21L858R). **(D)** The correlation between PFS and co-mutation TP53 (yes *vs.* no). PFS, progression-free survival; PD-L1, programmed death-ligand 1; 19DEL, exon 19 deletion; 21L858R, a substitution of lysine with arginine in exon 21; EGFR, epidermal growth factor receptor.

In this series, PFS events occurred in 19 out of 73 patients, with a median follow-up period of 9 months (95% CI, 7.0–11.0, data cutoff date of September 24, 2023). Of these patients, 49 achieved a PR, resulting in an ORR of 67.1% (49/73 patients, with a 95% CI ranging from 56.1% to 78.2%). The ORRs for patients with 19DEL or 21 L858R were 67.6% (95% CI, −51.7 to 83.4) and 64.6% (95% CI, 47.8–81.6), respectively (as indicated in **Table 2**). There were 32 patients who had brain metastases at the time of initial diagnosis. Of these, 26 had measurable and evaluable target lesions within the brain, and the intracranial ORR was 84.6% (95% CI, 69.8–99.5), with all 26 patients experiencing some degree of tumor reduction (**Table 3**). The estimated median PFS for all patients with EGFR-mutated NSCLC undergoing treatment with furmonertinib was 19.5 months (95% CI, 14.6–24.4 months), as shown in **Figure 2A**. The median intracranial PFS was 16 months (95% CI, 15.6–16.4 months), which is detailed in **Figure 2B**. We further explored the relationship between PFS and several factors: the presence of brain metastases, PD-L1 status (less than 1% *vs.* 1% or greater), EGFR mutation subtype (exon 19 deletion *vs.* 21L858R point mutation), and the presence of TP53 co-mutations. The analyses revealed no statistically significant associations between these factors (as presented in **Figures 3A–D**). As of the last data update, OS had not been reached due to immature data. In the univariate analysis, gender (p = 0.04) and liver metastasis (p = 0.03) showed a significant association with progression-free survival (PFS) (**Table 4**). However, other clinicopathological factors did not show any significant associations with PFS (all p > 0.05) (**Table 4**). In the multivariate Cox regression analysis model, which included all factors significantly associated with PFS from the univariate analysis, no factors were found to be significantly associated with PFS (**Table 4**).

**Table 2:** Best clinical response of patients receiving furmonertinib.

Best response	All patients (N = 73), n%	Patients with 19DEL mutations (N = 37), n%	Patients with 21L858R mutations (N = 34), n%
CR	0	0	0
PR	49 (67.1)	25 (67.6)	22 (64.7)
SD	21 (28.8)	11 (29.7)	10 (29.4)
PD	3 (4.1)	1 (2.7)	2 (5.9)
ORR	49 (67.1; 95% CI, 56.1–78.2)	25 (67.6; 95% CI, −51.7 to 83.4)	22 (64.7; 95% CI, 47.8–81.6)
DCR	70 (95.9, 95% CI, 91.2–100.6)	36 (97.3; 95% CI, −91.8 to 102.8)	32 (94.1; 95% CI, 85.8–102.5)

CR, complete response; PR, partial response; SD, stable disease; PD, progressive disease; ORR, objective response rate; DCR, disease control rate.

**Table 3:** Best intracranial clinical response for patients with brain metastases receiving furmonertinib.

Best intracranial response	All patients (N = 26), n%
CR	7 (26.9)
PR	15 (57.7)
SD	4 (15.4)
PD	0 (0)
ORR	22 (84.6; 95% CI, 69.8–99.5)
DCR	26 (100, 95% CI, 100–100)

CR, complete response; PR, partial response; SD, stable disease; PD, progressive disease; ORR, objective response rate; DCR, disease control rate.

**Table 4:** Univariate and multivariate Cox regression analyses of PFS.

Parameters	Univariate analysis		Multivariate analysis	
	*HR* (95% CI)	p-Value	*HR* (95% *CI*)	p-Value
Sex: male *vs.* female	1.26 (0.61–2.28)	0.41		
Age: <65 *vs.* ≥65	2.64 (1.05–6.65)	0.04		0.053
Smoking: yes *vs.* no	0.95 (0.33–2.74)	0.43		
ECOG PS: 0–1 *vs.* ≥2	2.17 (0.72–6.53)	0.16		
Mutation status 19DEL 21L858R Compound Rare		0.40		
Stage IVA *vs.* IVB	0.50 (0.12–2.04)	0.33		
Number of metastatic organs: <3 *vs.* ≥3	1.26 (0.42–3.79)	0.98	2.226 (1.313–3.773)	
Brain metastases: yes *vs.* no	0.71 (0.28–1.83)	0.48		
Liver metastases: yes *vs.* no	2.16 (0.28–16.43)	0.03		0.087
Bone metastases: yes *vs.* no	0.72 (0.30–1.76)	0.48		
Adrenal metastases: yes *vs.* no	2.26 (0.33–15.44)	0.41	1.855 (1.181–2.914)	

PFS, progression-free survival; ECOG PS, Eastern Cooperative Oncology Group performance status.

### Safety profiles

Adverse events were noted in 25 patients who received furmonertinib as a first-line treatment. Diarrhea (24%), anemia (36%), and liver injury (32%) were the most frequently reported adverse events. There were serious adverse events in four patients (16%): one experienced diarrhea, one had thrombocytopenia, another suffered liver injury, and the last had an increase in blood creatinine. There was only one case of grade 1 interstitial lung disease reported, and no patients stopped treatment due to adverse events. Additional adverse events are compiled in **Table 5**. Overall, the toxicity profile for furmonertinib-treated patients was manageable and largely aligned with that reported in previous studies.

**Table 5:** Adverse event profiles.

Event	Treated population (25 patients have documented side effects)	
	Any grade	Grades 3, 4, 5
Diarrhea	6/25 (24%)	1/25 (4.0%)
Rash	4/25 (16%)	0
Anemia	9/25 (36.0%)	0
Decreased white blood cell count	4/25 (16%)	0
Neutropenia	4/25 (16.0%)	0
Thrombocytopenia	4/25 (16%)	1/25 (4%)
Interstitial lung disease	1/25 (4.0%)	0
Liver injury (elevated ALT or AST)	8/25 (32.0%)	1/25 (4.0%)
Hypothyroidism	1/25 (4.0%)	0
Hypokalemia	3/25 (12%)	0
Hyperlipidemia	1/25 (4.0%)	0
Increased blood creatinine	1/25 (4.0%)	1/25 (4.0%)

ALT, alanine aminotransferase; AST, aspartate aminotransferase.

## Discussion

As far as we know, this is both the first and largest observational study conducted in China on using furmonertinib as an initial treatment in everyday clinical practice. Our research encompassed a diverse patient group, including those with poor PS, elderly individuals over the age of 75, patients with uncommon and complex EGFR mutations, and those with active brain metastases, including leptomeningeal involvement—populations typically not included in prospective and randomized clinical trials. The findings from our study validate the use of furmonertinib as a viable first-line treatment option for patients with EGFR mutations in real-world conditions, just as it has been shown in controlled clinical trials. Nonetheless, given the small sample size and the retrospective nature of our study, additional research is needed to confirm these results.

Our current study included 35 male patients displaying EGFR mutations, accounting for nearly half of the participants, thereby emphasizing the importance of molecular testing in NSCLC patients regardless of sex. This finding appears to contradict previous research that suggested a lower incidence of EGFR mutations in men (14, 15). Moreover, the study involved 32 patients (43.8%) with brain metastases at the time of their initial diagnosis, which is a higher incidence compared to what has been reported in clinical trials (9). This greater prevalence of brain metastases in a real-world setting could potentially affect the survival outcomes observed in this study. An additional noteworthy detail of our report is that all included patients were diagnosed with stage IV cancer, unlike the population in the FURLONG trial. Our study found that the median PFS was 19.5 months, which is less than what was documented in the FURLONG study. Possible explanations for this shorter PFS may include the inclusion of a higher number of male patients, more patients with brain metastases, and a greater number of cases at stage IV.

It is widely recognized that PD-L1 is a key biomarker for predicting responses to ICIs. Some studies have also indicated a correlation between PD-L1 expression and the effectiveness of EGFR-TKIs, though this theory remains the subject of debate (16–20). Quite a few studies have found no significant link between PD-L1 expression levels and the efficacy of EGFR-TKIs (21). In our study, we evaluated PD-L1 expression in 32 patients, finding that the majority were negative (PD-L1 < 1%), aligning with prior research suggesting that individuals with EGFR mutations tend to have low PD-L1 expression levels (22–24). Dong and colleagues compiled data from 15 studies, proposing that PD-L1 expression is inversely related to EGFR mutation status (25). Their investigation into the correlation between mRNA and PD-L1 protein levels in surgical samples from The Cancer Genome Atlas (TCGA) and an internal database (Guangdong Lung Cancer Institute (GLCI)) supported the notion that EGFR-wild type tumors have higher PD-L1 expression compared to EGFR-mutated tumors. Contrarily, there have been reports asserting the opposite (26). Thus, the association between PD-L1 expression and EGFR mutations remains a topic of debate. In our case, the data showed no differences in PFS when categorizing by PD-L1 levels. However, due to the small number of cases included in the study, these findings should be approached with caution.

Previous literature has indicated that the effectiveness of EGFR-TKIs can vary based on the type of EGFR mutation present (7–9). For instance, in the FLAURA China study, individuals with EGFR 19DEL achieved a longer PFS than those with the L858R point mutation in exon 21 (27). Similarly, the AENEAS study showed that patients with the 19DEL mutation had a more favorable response compared to those with the 21L858R mutation, despite achieving comparable benefits to the control group (8). The findings of the FURLONG study echo these observations, suggesting that patients with the 19DEL mutation could be considered a subgroup with a potentially better prognosis compared to those with the 21L858R mutation (9). These results have prompted some experts to propose that patients with 19DEL and 21L858R mutations might benefit from distinct therapeutic approaches. However, in our current research, we observed no significant difference in PFS between patients with 19DEL and 21L858R mutations. The discrepancy with the FURLONG study’s results could be due to the limited sample size in our study. Looking to enhance the efficacy of EGFR-TKIs for patients with 21L858R mutations, some researchers have explored the combination of EGFR-TKIs with anti-angiogenesis drugs. Various studies have suggested that combining first-generation EGFR-TKIs with vascular endothelial growth factor (VEGF) inhibitors might extend PFS for patients with the 21L858R mutation to levels similar to those with the 19DEL mutation (28–31). Nevertheless, intriguingly, the addition of VEGF inhibitors does not seem to improve the effectiveness of osimertinib, which is a third-generation EGFR-TKI (32–34). The question of whether first-generation EGFR-TKIs combined with VEGF inhibitors are superior to monotherapy with third-generation EGFR-TKIs for patients with EGFR-sensitive mutations remains open for investigation. Nevertheless, third-generation EGFR-TKIs are currently the preferred treatment option for patients with such sensitive mutations.

In both univariate and multivariate analyses, we observed that PFS was not associated with Eastern Cooperative Oncology Group (ECOG) performance status, the number of metastatic sites, the presence of brain or liver metastases, or other factors. This finding differs from that of previous studies. A possible reason for this discrepancy could be the limited number of cases and the retrospective nature of our study.

Our research has several limitations. First, the follow-up period may not be adequately long, which could introduce bias into our conclusions. Also, due to the limited duration of observation, the OS data were not mature. We plan to provide an update on OS once sufficient events have occurred and an appropriate follow-up duration has been reached. Second, our study was retrospective and included a limited number of cases. Potential concerns should be noted: efficacy evaluations were performed according to RECIST 1.1 by investigators, which could lend toward more objective outcomes. Therefore, further research is necessary.

## Conclusions

In conclusion, our study suggests that furmonertinib could be a preferred treatment option as a first-line therapy for patients with EGFR-sensitive mutations. We observed comparable PFS in the real-world setting relative to that in randomized clinical trials. These findings underscore the potential of furmonertinib as a viable choice in real-world clinical practice.

## Data availability statement

The original contributions presented in the study are included in the article/supplementary material. Further inquiries can be directed to the corresponding authors.

## Ethics statement

The studies involving humans were approved by the Ethics Committee of the First Affiliated Hospital of Zhengzhou University. The studies were conducted in accordance with the local legislation and institutional requirements. Current work was retrospective study and individuals’ information will not be shared, hence, the Ethics Committee of the First Affiliated Hospital of Zhengzhou University exempted the informed consent.

## Author contributions

NY: Conceptualization, Data curation, Formal analysis, Funding acquisition, Investigation, Project administration, Resources, Writing – original draft, Writing – review & editing. SG: Conceptualization, Data curation, Formal analysis, Methodology, Project administration, Resources, Writing – original draft, Writing – review & editing. SH: Formal analysis, Methodology, Resources, Writing – original draft, Writing – review & editing. HZ: Data curation, Formal analysis, Investigation, Methodology, Project administration, Supervision, Validation, Writing – original draft, Writing – review & editing. XL: Conceptualization, Data curation, Investigation, Methodology, Project administration, Resources, Software, Supervision, Validation, Visualization, Writing – original draft, Writing – review & editing.
