# Corrigendum: The efficacy of furmonertinib in untreated advanced NSCLC patients with sensitive EGFR mutations in a real-world setting: a single institutional experience

**DOI:** 10.3389/fonc.2024.1412472

**Published:** 2024-06-03

**Authors:** Ningning Yan, Sanxing Guo, Siyuan Huang, Huixian Zhang, Xingya Li

**Affiliations:** Department of Oncology, The First Affiliated Hospital of Zhengzhou University, Zhengzhou, Henan, China

**Keywords:** furmonertinib, non-small cell lung cancer, EGFR-mutated, epidermal growth factor, receptor, real-world setting

In the published article, there was an error in the legend for **Figure 2** as published. Furthermore, we have made the wrong graphical representation for **Figure 2**. The corrected figure and its legend appear below.

**Figure 2:**
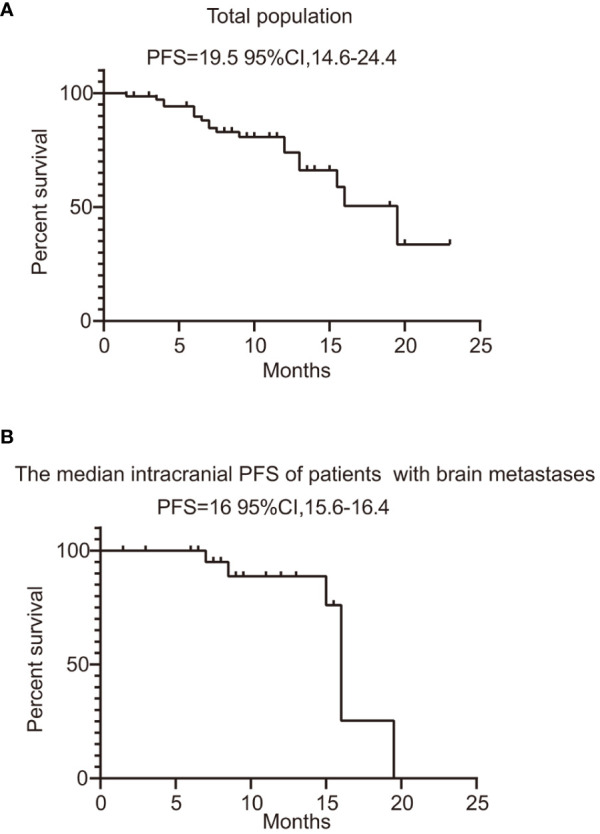
PFS of total population and patients with evaluable brain metastases. **(A)** PFS of all the patients treated with furmonertinib. **(B)** the median intracranial PFS of patients with brain metastases treated with furmonertinib. PFS, progression-free survival.

The authors apologize for this error and state that this does not change the scientific conclusions of the article in any way. The original article has been updated.

